# Vinegar-processed *Curcuma phaeocaulis* promotes anti-angiogenic activity and reduces toxicity in zebrafish and rat models

**DOI:** 10.1080/13880209.2021.1874427

**Published:** 2021-05-23

**Authors:** Wan Liao, Yi Chen, Zongping Zhu, Jiao Chen, Tianhui Gao, Boonjai Limsila, Yenchit Techadamrongsin, Lei Wang, Jiali Yu, Chaomei Fu, Rui Li

**Affiliations:** a State Key Laboratory of Characteristic Chinese Drug Resources in Southwest China, College of Pharmacy, Chengdu University of Traditional Chinese Medicine, Chengdu, P.R. China; b Department of Thai Traditional and Alternative Medicines, Institute of Thai-Chinese Medicine, Ministry of Public Health, Bangkok, Thailand; c Sichuan Provincial Orthopedic Hospital, China

**Keywords:** Volatile oil, LC_50_ values, intersegmental blood vessels, ovarian coefficient, uterine coefficient, cardiotoxicity, Ezhu

## Abstract

**Context:**

Processing with vinegar could enhance the efficacy and reduce the toxicity of *Curcuma phaeocaulis* Valeton. (Zingiberaceae), a Chinese herbal medicine with anti-inflammatory and antitumor activities.

**Objective:**

This study investigated the vinegar processing effects by evaluating anti-angiogenic effect and toxicity of *C. phaeocaulis* through zebrafish and rat models.

**Materials and methods:**

Zebrafish embryos (AB and FLk-GFP strain) were applied to evaluate toxicity, cardiotoxicity and anti-angiogenic activity of volatile oil, and water decoction of the raw and vinegar-processed *C*. *phaeocaulis.* Meanwhile, a blood stasis syndrome rat model was applied to study the toxicity by measuring the ovarian and uterine coefficient.

**Results:**

*Curcuma phaeocaulis* volatile oil and its vinegar-processed products in zebrafish had an LC_50_ of 67.315 and 95.755 μg/mL, respectively. *Curcuma phaeocaulis* water decoction and its vinegar-processed products had an LC_50_ of 161.440 and 206.239 μg/mL, respectively. The toxicity of vinegar-processed products was significantly lower than the raw, and the development characteristic of zebrafish embryos at different times confirmed these results. The volatile oil of vinegar-processed products could inhibit the growth of intersegmental blood vessels at the dose of 20 μg/mL, while the raw materials did not exhibit such effect at the same concentration. The rat experiment also confirmed that the volatile oil could reduce toxicity of ovarian and uterine.

**Discussion and conclusions:**

The study indicated that processing using vinegar could decrease toxicity and increase anti-angiogenic activity of *C. phaeocaulis*, which could be applied for clinical treatment. Further in-depth study on the synergism and detoxification mechanism of vinegar processing technology is needed.

## Introduction

Chinese herbal medicines (CHMs) have historically been utilised in China for the maintenance and treatment of various health conditions. Chinese medicinal processing is one of the most important pharmaceutical techniques that transforms medicinal raw materials into decoction pieces based on the theory of CHMs, which can influence dispensing, preparation and clinical applications (Chinese Pharmacopoeia Commission 2015; Wu et al. 2018). Various processing adjuvants are usually added to herbs, including vinegar, honey, wine, brine, ginger juice, bran, and rice. Adjuvants could change therapeutic effects and reduce drug toxicity, thereby facilitating the use of CHMs for specific needs in the clinic (Zhao et al. 2010; Jiang et al. 2013). Vinegar is an auxiliary material, and processing with vinegar is one of the essential processing methods which was first recorded in Recipes for Fifty-Two Ailments (*Wushi'er Bingfang* in Chinese) in the Western Han Dynasty, and this method has played an increasingly important role in promoting the development of traditional Chinese medicine (Wang et al. 2009). In the theory of CHMs, processing with vinegar possesses the effect of soothing the liver, relieving depression, preventing blood stasis, relieving pain, and reducing the toxicity (Liao et al. 2013, 2014). This processing is widely used in the preparation of many herb decoctions, such as Curcumae Rhizoma (Ezhu in Chinese), Bupleuri Radix (Chaihu), Kansui Radix (Kansui) and Phytolacca Radix (Shanglu).

*Curcuma phaeocaulis* Valeton (Zingiberaceae), is one of the best known medicinal materials in Sichuan Province, China (Yang et al. 2005). Traditionally, its rhizomes are frequently used in the clinic based on its blood circulation-promoting and pain-alleviating properties (Sasaki et al. 2003). *Curcuma phaeocaulis* processed with vinegar has been applied in clinics for thousands of years, and its vinegar processed slice is officially listed in the current China Pharmacopoeia. Previous studies (Li et al. 2011, 2016, 2019) have revealed that *C. phaeocaulis* possesses anti-inflammatory activity (Hao et al. 2014; Hou et al. 2015). The methanol extract of this plant could alleviate paw swelling and decrease serum haptoglobin concentrations in arthritis in mice (Tohda et al. 2006), as well as impart an antioxidant effect (Naik et al. 2011; Hao et al. 2014). Also, *C. phaeocaulis* has been reported to possess antitumor activity (Sasaki et al. 2003). It inhibits MCF-7 cell proliferation by inducing apoptosis, which is mediated by increasing ROS formation, decreasing mitochondrial membrane potential, regulating Bcl-2 family protein expression, and activating caspases (Chen et al. 2011).

An approved therapeutic option for the prevention of platelet aggregation could resort to vinegar-processed *C. phaeocaulis* (Mao et al. 2000). Intriguingly, our previous study also found that the effectiveness of *C. phaeocaulis* has increased and its toxicity has decreased after processed with vinegar, particularly its effect on treat the stagnation of vital energy and blood stasis (Liao et al. 2013). Conspicuously, the vinegar-processed *C. phaeocaulis* have long been used in traditional Chinese medicines for blood stasis sydrome. Nevertheless, application of vinegar-processed *C. phaeocaulis* in clinic lasts for more than a millennium owing to the multiple pharmaceutical effects, limited knowledge is available on underlying mechanisms of enhancing the therapeutic efficacy and reducing the toxicity after vinegar-processing. In our study, we adopted zebrafish as animal model to evaluate the toxicological (Ding et al. 2015; Yan et al. 2016) and anti-angiogenic (Alex et al. 2010; He et al. 2011) effect. The different effects of raw *C. phaeocaulis* and its vinegar-processed products on the embryo, heart, and blood vessels were also investigated. Subsequently, we utilised sexually mature female rats with blood stasis syndrome as another animal model. The body weight, ovary wet weight, ovary coefficient, uterus wet weight, and uterus coefficient of these indexes related to toxicity were measured to determine changes in the toxicity of raw *C. phaeocaulis* and its vinegar-processed products.

## Materials and methods

### Chemicals and reagents

Ten batches of *C. phaeocaulis* rhizome were collected from different areas of Sichuan Province from 2018 to 2019, and their vinegar-processed samples were prepared accordingly. All the samples were identified as the rhizomes of *C. phaeocaulis* by Professor Xianming Lu and were deposited in the School of Pharmacy, Chengdu University of Traditional Chinese Medicine. Reference standards of bisdemethoxycurcumin, demethoxycurcumin, curcumin, curdione, curcumol, germacrone, and β-elemene were purchased from Sichuan Weikeqi Biological Technology Co., Ltd. (purity > 98%; Sichuan, China). Methyl cellulose was purchased from Sigma-Aldrich (USA). All HPLC grade solvents were obtained from Fisher Scientific (Pittsburgh, PA, USA). Adrenaline hydrochloride injection was purchased from Tianjin Jinyao Pharmaceutical Co., Ltd. Compound Danshen Tablets were purchased from Yunnan Baiyao Group Co., Ltd. (Production Lot ZLC1434).

### Animals

Adult male and female, AB strain, and green fluorescent-labeled transgenic flk1-GFP zebrafish, weighing 0.25 ± 0.04 g (mean ± SEM) were obtained from Beijing Aisheng Biological Technology Co. Ltd. and bred at the Chengdu University of TCM using a zebrafish experimental platform. Zebrafish, at a 1:1 female to male ratio, were maintained in an aquarium with an automatic filtration system and covered with blue contact paper to reduce stress. The animal room was illuminated on a 14/10 h light/dark cycle, and room temperature was maintained at 25 ± 1 °C.

Female specific-pathogen-free Sprague Dawley (SD) rats (weight: 200 ± 20 g, six to eight-week-old) at sexual maturity were supplied by Chengdu Dashuo Experimental Animals Co., Ltd. (Sichuan, China). Animal welfare and experimental procedures were strictly conducted in accordance with the Guide for the Care and Use of Laboratory Animals and the related ethical regulations of Chengdu University of TCM (CDUTCM, permit CDU2019S121). The rat experiments used in this study were performed in compliance with the Guidelines for the Care and Use of Laboratory Animals published by the U.S. National Research Council (8th edition, National Academies Press 2011). The zebrafish received humane care according to the criteria of the National Institutes of Health and the Georgia Institute of Technology Institutional Animal Care and Use Committee.

### Sample preparation

Vinegar-processed *C. phaeocaulis* was prepared by the patented process, ‘The preparation of vinegar-processed *C. phaeocaulis*, ZL 2013 1 0400255.2’ (Liao et al. 2015). The detailed vinegar process is put the 30% 9° rice vinegar into suitable *C. phaeocaulis* for moistening 2 h and cook it until the rhizome of *C. phaeocaulis* is almost dry. This drying process includes evaporation at 120 °C for 30 min and cooling down.

The water decoction was obtained by the following methods: the samples of *C. phaeocaulis* and its vinegar-processed products (100 g each) were placed in containers and mixed with water at 15 times the volume. The samples were soaked for 30 min and decocted for 0.5 h twice. The extracted solutions were pooled together and the final volume was adjusted to 100 mL. The liquid was heated to 37 °C before administration. Volatile oil was obtained by the following methods: the samples of *C. phaeocaulis* and its vinegar-processed products (100 g each) and water (8 times content volume) were added, soaked for 1 h, and steam-distilled for 2–3 h. The volatile oil was dissolved in DMSO.

For quality control of vinegar-processed *C. phaeocaulis*, based on our previous reports (Liao et al. 2013, 2014; Gao et al. 2017), the levels of seven major constituents of the water decoction including bisdemethoxycurcumin, demethoxycurcumin, curcumin, curdione, curcumol, germacrone, and β-elemene, were determined by HPLC using a Zorbax SB-C_18_ (150 × 4.6 mm, 5 μm) column. The contents of bisdemethoxycurcumin, demethoxycurcumin, curcumin, curdione, curcumol, germacrone, and β-elemene were 0.00232, 0.05966, 0.2724, 0.3869, 1.2613, 0.7293, and 1.4212 mg/g, respectively. All the index compounds in vinegar-processed *C. phaeocaulis* samples in this study met the demand of the Pharmacopoeia of the People’s Republic of China, 2015 Edition. The detail data for quality control can be seen in the Supplementary Materials.

### Preparation of sample solution

Formaldehyde (100 mL) was dissolved in 900 mL of distilled water and 4 g of sodium dihydrogen phosphate. Disodium hydrogen phosphate (6.5 g) was added to the solution. The pH was adjusted to 7.0 and stored in a closed container. Danshen tablets were ground to a powder, and a 0.5 g/kg solution was prepared using normal saline and stored at 4 °C.

### Zebrafish toxicity assay

To study the LC_50_ values of lethal toxicity research, zebrafish embryos (AB strain, 12-hpf) were randomly placed in a 24-well plate, with six embryos in each well, and each dose treatment consisted of two replicates. *Curcuma phaeocaulis*, its volatile oil, and water decoction were used as treatments using various dosages (25, 50, 100, 200, and 400 µg/mL) for 12 h. A blank control group and a solvent control group (containing 0.5% DMSO) were also employed. The original culture medium was drawn, and 750 µL of the working solution were added to each well and kept in a constant-temperature incubator until hatching. The mortality rate of each dose was recorded, and LC_50_ values were determined using a LEICA M165FC stereo microscope after 12 h. The embryos were monitored once every 12 h by placing the embryos under the slides (with a glass tank) and were fixed with 4% methyl cellulose. Photomicrographs of the embryos were collected using a Zeiss Inversed Fluorescent Microscope-Observer A1.

To record the heartbeat frequency of each dose group, zebrafish embryos (AB strain, 48-hpf) were randomly loaded into a 24-well plate, with six embryos in each well, and each dose was consisted of two replicates. The *C. phaeocaulis* and its vinegar-processed products of volatile oil and water decoctions were administered as different doses (10, 20, 50, 100, 200 µg/mL). A blank control group containing 0.5% DMSO was used. The working solution (750 µL) was added to each group. The heartbeat frequency of each dose group was recorded using a LEICA M165FC fluorescence microscope after 12 h and 24 h. After 12 h, different dose group deciduate embryos were placed under the slides (with a glass tank) and fixed with 4% methyl cellulose. The zebrafish position was adjusted to make both eyes and the somite overlap, and the tail and body were placed on the same horizontal plane. Photographs were collected using a stereoscopic microscope.

### Zebrafish vessel anti-angiogenesis assay

Zebrafish embryos (FLk-GFP strain, 24-hpf), a targeted animal model for the study of angiogenesis were randomly loaded into a 24-well plate, with six embryos in each well, and each dose consisted of two duplicates. The PTU was employed as a blank control, and *C. phaeocaulis* vinegar-processed volatile oil was added to each well using different concentrations (10, 20, 50, 100, 200 µg/mL). The working solution (750 µL) was added to each group, and the plate was placed in a constant temperature incubator for hatching. After 12 h, fluorescein angiography growth was observed under a Zeiss Inversed Fluorescent Microscope-Observer A1.

### Changes in toxicity of raw *C. phaeocaulis* and its vinegar-processed products in female rats at sexual maturity

All animals were fed in an environmentally-controlled breeding room (temperature maintained at about 25 °C with a 12 h light/dark cycle) for at least one week before starting the experiments and fed with standard laboratory food and water *ad libitum*. All rats were randomly divided into seven groups of 10 rats each as follows: control group, control + raw group (CR), control + vinegar group (CV), model group (blood stasis syndrome model), model + raw group (MR), model + vinegar group (MV), model + compound Danshen tablet group (positive drug control group, MD).

The model groups received a hypodermic injection of 0.1% epinephrine hydrochloride every 4 h for a total of 3 times on the first day, then the rats were placed in 0 °C to 2 °C ice water twice at 4 min intervals. On the second and third days, these rats received a hypodermic injection of 0.1% epinephrine hydrochloride every 4 h for a total of 2 times and then were placed in 0 °C to 2 °C ice water. After the next successful moulting, five groups, including the CR group, CV group, MR group, MV group, and MD group, received raw medicine (5.6 g/kg/day), vinegar products (5.6 g/kg/day), or compound Danshen tablets (0.5 g/kg/day) via intragastric administration. The control and model groups were administered the same volume of normal saline for 18 d. The rats were housed in an animal room in a controlled condition (22–24 °C) with free access to normal food and de-ionized water. All groups of rats were in a healthy condition before intragastric administration.

After the rats were anaesthetised and sacrificed with pentobarbital sodium, the uterus and ovaries were immediately collected, and the surrounding connective tissues and lipids were carefully removed. The weight and the coefficient of the uterus and the ovary were calculated. After weighing one side of the uterus and the ovaries, these were stored in a −80 °C freezer, whereas the other side of the uterus was fixed in 10% formalin.

Uterine coefficient = Uterine wet weight (g)/Rat body weight (g) × 100%

Ovarian coefficient = Ovarian wet weight (g)/Rat body weight (g) × 100%

### Statistical analysis

All data were analysed by the software SPSS 19.0 and Origin 7.5, and LC_50_ values were determined using the method of Probit. Heart rate was determined as the average value ± standard deviation (x ± s). The *t*-test was used to compare two groups, and ANOVA was used to compare all groups. Differences with a *p <* 0.05 were deemed statistically significant.

## Results

### Acute toxicity and cardiotoxicity of *C. phaeocaulis* before and after vinegar processing in a zebrafish model

**Figure 1.**
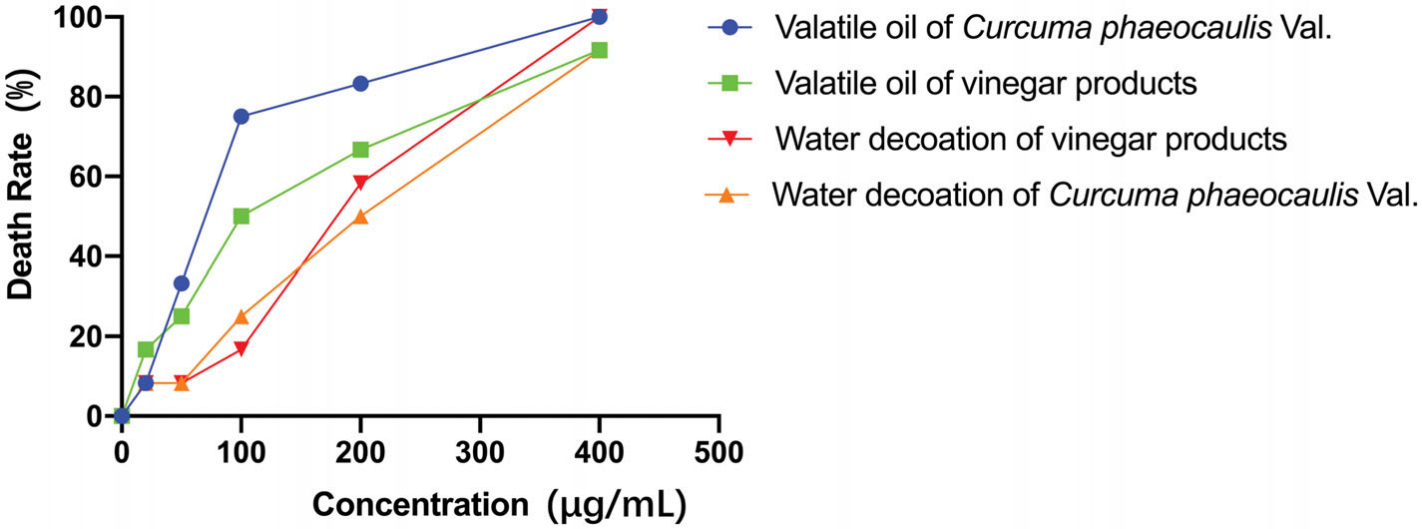
The toxicity *C. phaeocaulis* before and after processing with vinegar in zebrafish.

**Figure 2.**
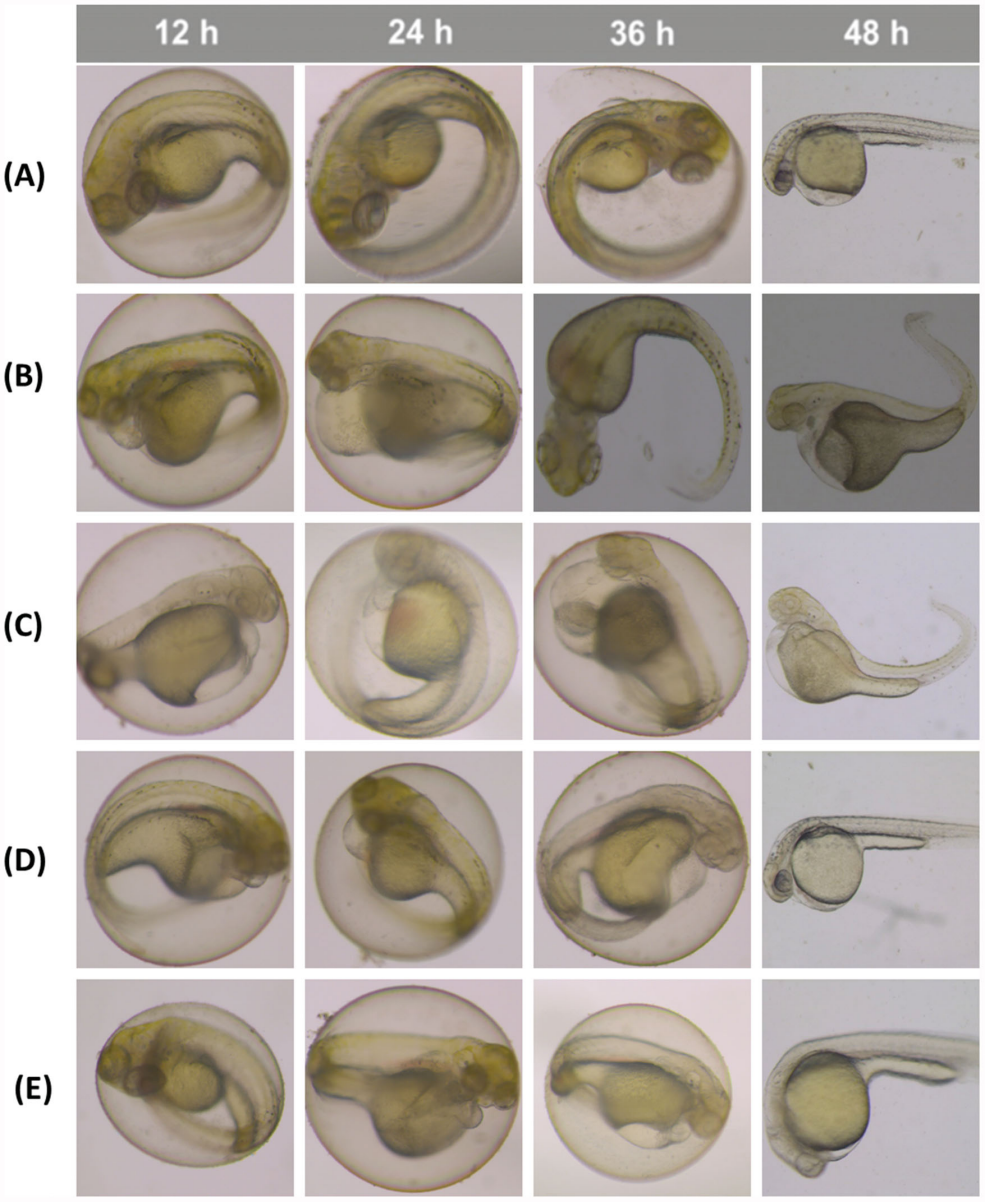
Embryonic development of zebrafish at different time points. (A) Blank control group; (B) Volatile oil of *C. phaeocaulis* (100 µg/mL). (C) Volatile oil of *C. phaeocaulis* vinegar-processed products (100 µg/mL); (D) Water decoction of *C. phaeocaulis* (200 µg/mL); (E) Water decoction of *C. phaeocaulis* vinegar-processed products (200 µg/mL).

**Figure 3.**
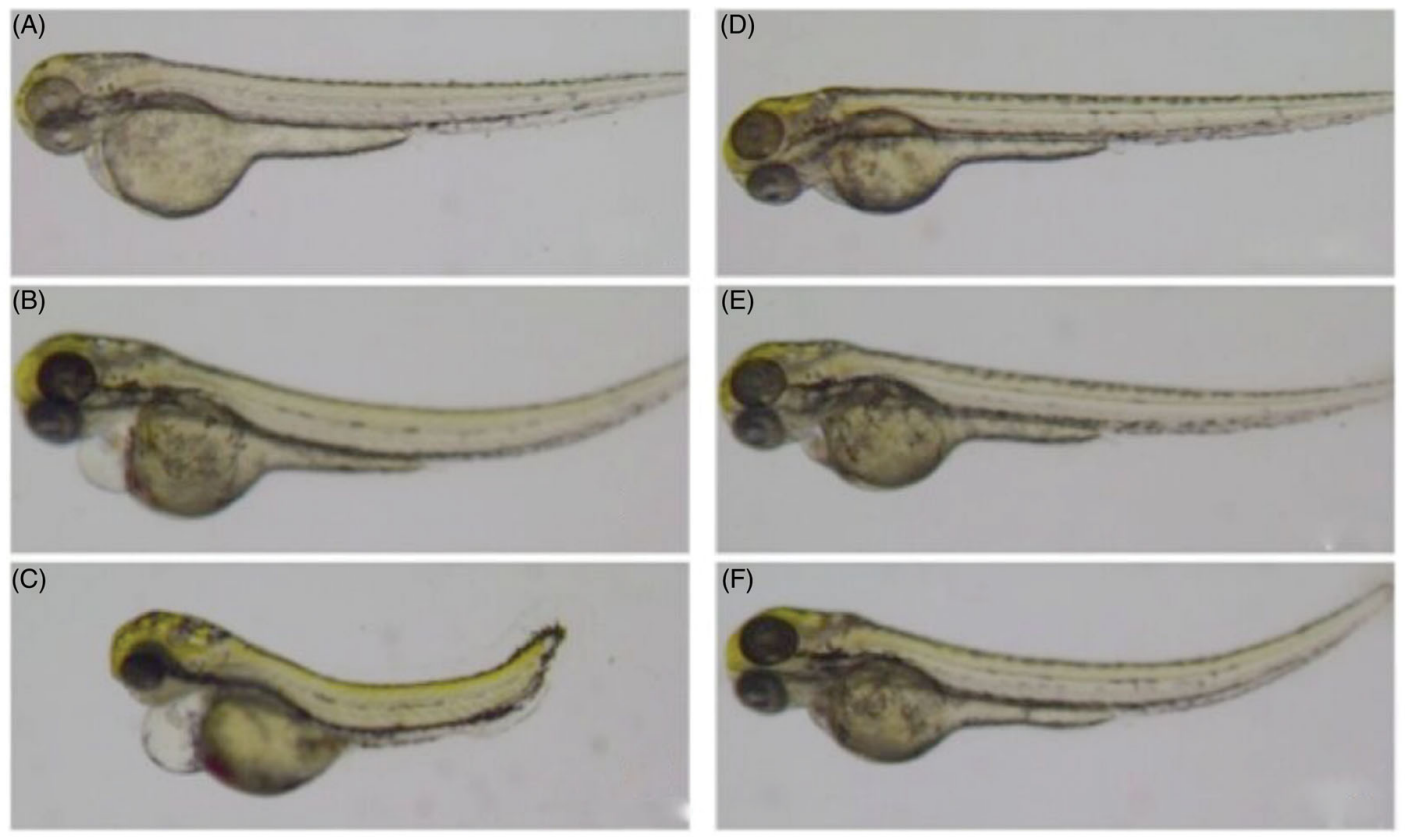
The effect of volatile oil of *C. phaeocaulis* and its vinegar-processed products on zebrafish heart morphology. (A) Blank control group. (B) Volatile oil of *C. phaeocaulis* (20 µg/mL). (C) Volatile oil of *C. phaeocaulis* (50 µg/mL). (D) Blank control group. (E) Volatile oil of *C. phaeocaulis* vinegar-processed products (20 µg/mL). (F) Volatile oil of *C. phaeocaulis* vinegar-processed products (50 µg/mL).

The 0.5% DMSO aqueous solution showed no obvious toxicity in zebrafish. The LC_50_ values of volatile oil of *C. phaeocaulis* and its vinegar-processed products in zebrafish were 67.315 and 95.755 µg/mL, respectively. The LC_50_ values of the water decoction of *C. phaeocaulis* and its vinegar-processed products were 161.440 and 206.239 µg/mL, respectively. Our results showed that the toxicity of vinegar-processed products was significantly lower than that of raw *C. phaeocaulis* in forms of both volatile oil and water decoction; the toxicity of *C. phaeocaulis* and its vinegar products water decoctions were dramatically lower than volatile oil (Figure 1). Furthermore, Figure 2 showed that the volatile oil of *C. phaeocaulis* has teratogenic effects on zebrafish embryos, there have been varying degrees of scoliosis, pericardial edoema, less melanin phenomenon, and the similar concentrations of raw products volatile oil have more serious trend on teratogenic effects embryos than the vinegar products. Therefore, our results also comfirmed that the vinegar-processed products induced lower toxicity than raw *C. phaeocaulis* through comparing the development characteristic of zebrafish embryonic at different time points. In addition, The raw products volatile oil of *C. phaeocauli*s trend on zebrafish cardiac toxicity was the most obvious, emerging the heart membrane bleeding, blood cells accumulate in the heart area phenomenon, the trend of the increase of the heart rate and the time of the function of the time were obviously decreased (Figure 3). And the cardiotoxicity of zebrafish embryos was most prominent when the dose of volatile oil of *C. phaeocaulis* was 20 and 50 µg/mL, respectively. While the volatile oil of vinegar products has no obvious toxic effect on the heart. Therefore, our results showed that processing using vinegar could reduce the toxicity of *C. phaeocaulis* in a zebrafish model.

### Anti-angiogenesis of *C. phaeocaulis* before and after processing with vinegar in a zebrafish model

**Figure 4.**
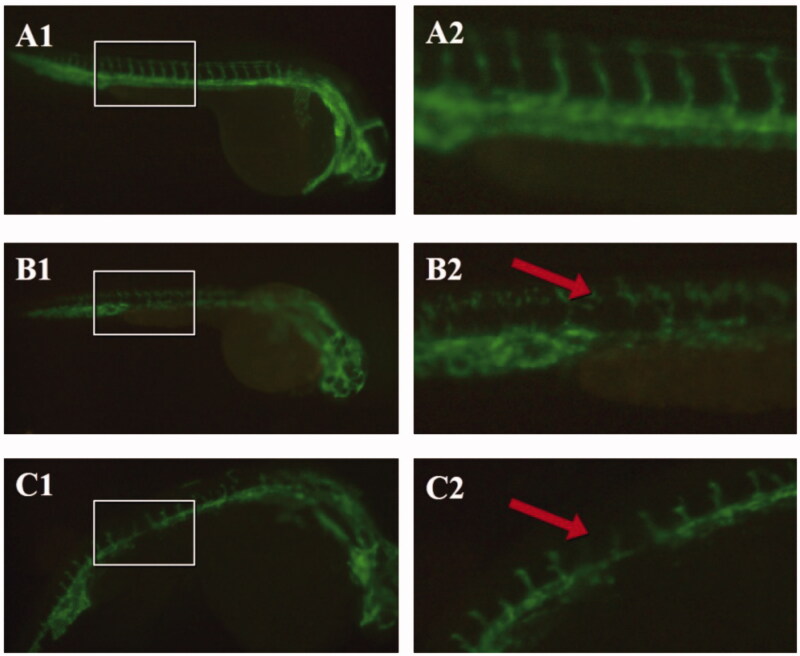
Vascular fluorescence of flk-GFP zebrafish strains. A. Blank control group; B. Volatile oil of *C. phaeocaulis* (20 µg/mL); C. Volatile oil of *C. phaeocaulis* vinegar-processed products (20 µg/mL). (A1-C1:×50, A2-C2:×100).

The volatile oil of *C. phaeocaulis* and its vinegar products could inhibit intersegmental blood vessels of 24-hpf zebrafish embryos after 12 h of treatment using a dose of 20 µg/mL without embryo teratogenic and toxicity, indicating that this dose could inhibit intersegmental blood vessels but not induce abnormalities in the zebrafish embryos. Figure 4 shows that at a dose of 20 µg/mL, the intersegmental blood vessels of the group of volatile oil of vinegar-processed products became missing and disorderly, the main blood vessel was broken and obviously missed which indicated that the volatile oil of vinegar-processed products could significantly inhibit intersegmental blood vessels, whereas the group treated with volatile oil of *C. phaeocaulis* exhibited slight inhibition. The application of *C. phaeocaulis* processed with vinegar resulted in an increase in anti-angiogenesis in the zebrafish model. Collectively, these results show that vinegar processing enhances the anti-angiogenesis effect of *C. phaeocaulis*.

### Change in the toxicity of raw *C. phaeocaulis* and its vinegar-processed products in female rats at sexual maturity

#### Weight measurement

During the administration period, the general condition and mental status of the rats in each experimental group were not as active as the control group during the first day. There was no obvious difference in the intake and drinking of water of each group compared to the control group.

Compared to the control group during the post-drug period, the body weights of the rats were distinctly different in the CR and CV groups (*p < 0.05). The weight of the model group was significantly lower than the control group (**p < 0.01). Compared to the model group, the body weights of the rats in each treatment group during the experimental period were statistically significant, ^Δ^*p* < 0.05, and ^ΔΔ^*p* < 0.01. There was no significant difference between CR and CV, MR and MV, CR and MR, and CV and MV groups (Table 1).

**Table 1.** Changes in the body weights of rats (*n* = 8).

Group	Dose (g/kg)	Original body weight	Day 1	Day 4	Day 7	Day 10	Day 13	Day 16
Control	0	210.98 ± 1.59	222.21 ± 8.23	220.98 ± 14.30^ΔΔ^	228.01 ± 12.33^ΔΔ^	224.31 ± 14.29	231.86 ± 10.17	234.26 ± 14.49
CR	5.6	203.93 ± 3.99	205.54 ± 10.66**^Δ^	217.83 ± 9.31^ΔΔ^	222.23 ± 12.03^ΔΔ^	209.03 ± 21.49*	227.35 ± 19.25*	231.01 ± 15.87
CV	5.6	207.03 ± 2.78	210.84 ± 11.43	214.43 ± 12.30^Δ^	212.53 ± 7.33*^ΔΔ^	201.81 ± 7.53**^Δ^	218.15 ± 8.02*	228.33 ± 7.71^Δ^
Model	0	210.45 ± 7.69	218.61 ± 13.72	199.70 ± 17.05**	192.84 ± 22.54**	220.23 ± 18.20	223.56 ± 15.35	231.60 ± 13.95
MR	5.6	204.67 ± 6.13	208.73 ± 14.76*	216.73 ± 13.19^Δ^	221.05 ± 10.41^ΔΔ^	226.44 ± 16.02	210.82 ± 10.12**	232.20 ± 8.83
MV	5.6	200.43 ± 2.37	202.44 ± 14.74**^Δ^	213.39 ± 13.14^Δ^	219.43 ± 14.22^ΔΔ^	223.00 ± 16.65	226.41 ± 20.60	239.10 ± 16.45
MD	0.5	211.11 ± 5.82	224.86 ± 11.39	233.80 ± 13.77^ΔΔ^	235.96 ± 12.15^ΔΔ^	237.71 ± 11.65^Δ^	242.28 ± 12.57^Δ^	244.05 ± 16.21

*p < 0.05 and **p < 0.01 compared to the normal control group; ^Δ^*p* < 0.05 and ^ΔΔ^*p* < 0.01, compared to the model control group. Control + raw group (CR), control + vinegar group (CV), model group (blood stasis syndrome model), model + raw group (MR), model + vinegar group (MV), model + compound Danshen tablet group (positive drug control group, MD).

### Changes in ovarian wet weight and measurement of ovarian coefficient in rats

Compared to the control group, the ovary wet weights and ovary coefficient of each experimental group were higher. There was a significant difference in the wet weights and ovary coefficient of the ovaries among the CR, CV, MR comparing with control groups (*p < 0.05). The wet weight and ovary coefficient of the ovaries of the model group was significantly higher than in the control group (*p <* 0.01). Compared to the model group, the ovary weight and ovary coefficient of the control group was markedly lower than the model group, *p* < 0.01, whereas that of the ovary weight of MD group was significantly higher than the model group (*p* < 0.05), the ovary coefficient of the MD groups were significantly lower than the model group (*p* < 0.01) (Table 2).

**Table 2.** Changes in wet weights and coefficients of ovary and uterus in rats (*n* = 8).

Group	Ovary wet weight (g)	Ovarian coefficient (%)	Uterus wet weight (g)	Uterine coefficient (%)
Control	0.0098 ± 0.0035^ΔΔ^	0.0514 ± 0.0021^ΔΔ^	0.5979 ± 0.1690	0.2716 ± 0.0770
Model	0.0226 ± 0.0080**	0.0643 ± 0.0113*	0.6166 ± 0.2813	0.2836 ± 0.1284
CR	0.0193 ± 0.0068*	0.0661 ± 0.0125*	0.4829 ± 0.1574	0.2276 ± 0.0661
CV	0.0234 ± 0.0083*	0.0695 ± 0.0089**	0.4978 ± 0.2478	0.2334 ± 0.1157
MR	0.0159 ± 0.0056*	0.0615 ± 0.0119	0.5479 ± 0.2068	0.2505 ± 0.0918
MV	0.0294 ± 0.0104	0.0649 ± 0.0079*	0.5638 ± 0.2524	0.2570 ± 0.1217
MD	0.0414 ± 0.0146^Δ^	0.0531 ± 0.0185^ΔΔ^	0.6600 ± 0.2645	0.2891 ± 0.1333

Compared to the normal control group, *p < 0.05, **p < 0.01; compared to the model control group, ^Δ^*p* < 0.05, ^ΔΔ^*p* < 0.01. Control + raw group (CR), control + vinegar group (CV), model group (blood stasis syndrome model), model + raw group (MR), model + vinegar group (MV), model + compound Danshen tablet group (positive drug control group, MD).

### Measurement of uterine wet weight and uterine coefficient

The uterine wet weight and uterine coefficient were statistically different among groups. There was a slight difference in the wet weights of the uterine among the CR, CV, and control groups. Compared to the model group, the uterine weight of the control group was markedly lower than the model group, whereas that of the MD group was significantly higher than the model group. The uterine coefficient of the model group was markedly different from the control group. In the control groups and model groups, the wet weights of the uterine among the CR are lower than CV, respectively (Table 2).

## Discussion

Recent studies have indicated that Qi-invigorating and blood-breaking medicines such as *C. phaeocaulis* could reduce the concentration, viscosity, and cohesion of blood, and inhibit intersegmental blood vessels selectively as well as improve microcirculation. The zebrafish model was used to explore the toxicity and determine the best dosage that could be administered during gestation, due to this species is highly homologous to human beings (87%), the transparency of the embryo, easy manipulation, short testing period, and small amount of testing drugs required are typical reasons for using this model. Our zebrafish embryo experiments found that the occurrence of scoliosis, pericardialites, melanotic pigment significantly decreased after treatment with 100 µg/mL volatile oil of *C. phaeocaulis* and its vinegar-processed products. Teratogenesis of volatile oil of *C. phaeocaulis* was more apparent than that of the volatile oil of its vinegar-processed products at the same dose. However, no abnormalities were observed using the same dose of the water decoction of this plant and its vinegar-processed products. Only a dose of the water decoction of *C. phaeocaulis* and its vinegar-processed products at 200 µg/mL resulted in zebrafish embryos with scoliosis, pericardialites, and melanotic pigment decrease, and the symptoms were weaker than that using 100 µg/mL volatile oil. Therefore, the present findings suggested that the toxicity of the water decoction was lower than that of the volatile preparation. *Curcuma phaeocaulis* before and after processing with vinegar was also toxic to zebrafish embryos and hearts, and the toxicity of the vinegar-processed products was lower than that volatile oil of *C. phaeocaulis*. These findings indicate that processing with vinegar could reduce the toxicity in embryos. We have also determined that 24-hpf is a better intervention period for the study of anti-angiogenesis of *C. phaeocaulis* before and after processing with vinegar in the zebrafish model. The optimised dose of vinegar-processed products or raw medicinal materials was 20 µg/mL, which could inhibit intersegmental blood vessels and maintain normal zebrafish embryonic activities. After processing with vinegar, the volatile oil of *C. phaeocaulis* at a low concentration significantly inhibited intersegmental blood vessels.

In the experiment of changes in toxicity of raw *C. phaeocaulis* and its vinegar-processed products in female rats at sexual maturity, we established a blood stasis syndrome model based on earlier investigations. Noteworthily, we found that, compared to the control, the rats in each group showed lower weight gain, except for the model + vinegar -processed group, which showed no significant difference in body weight. Compared to the control, the wet weight and ovarian coefficients of model control and model treatment groups that contained *C. phaeocaulis* were significantly higher, whereas there was no significant difference in the vinegar -processed *C. phaeocaulis* group and other *C. phaeocaulis* groups. These experimental results indicate that the effect of *C. phaeocaulis* on control rats group was significantly higher than on the model rats, and the curative effect of raw *C. phaeocaulis* was better than the processed product. However, to establish guidelines for clinically safe medication, further studies are warranted. The effects of *C. phaeocaulis* in normal mature female rats and blood stasis syndrome were investigated, and the safety of clinical drugs was guided by the idea of ‘no death.’ The toxicity of zedoariae was also investigated. In conclusion, the present studies demonstrated that processing with vinegar could alleviate toxicity, and enhance anti-angiogenic activity. This study provides preliminary evidence for the effect of vinegar-processed *C. phaeocaulis* and CHMs processing.

## Conclusions

This study showed that the vinegar-processed products induced lower toxicity than raw *C. phaeocaulis* in a zebrafish model and rat model with blood stasis syndrome based on various physiological indices. Meanwhile, the volatile oil extracted from vinegar-processed products could inhibit the growth of intersegmental blood vessels, while the raw materials did not show such effects at the same concentration. Our findings confirmed the effects of *C. phaeocaulis* with reducing toxicity and preventing blood stasis after vinegar processed. Moreover, it laid a foundation for further animal experiments with larger sample size, as well as for in-depth study on the mechanism of synergism and detoxification.

## Supplementary Material

Supplement Material
