# Supplementary material for: Vinegar-processed Curcuma phaeocaulis promotes anti-angiogenic activity and reduces toxicity in zebrafish and rat models
Source: Pharm Biol. 2021 May 23;59(1):408–15. doi: 10.1080/13880209.2021.1874427 (PMC8159270; doi:10.1080/13880209.2021.1874427)
Supplement: Supplement Material [file IPHB_A_1874427_SM1540.docx]

**Vinegar-processed *Curcuma* *phaeocaulis* promotes anti-angiogenic activity and reduces toxicity in zebrafish and rat models**

Wan Liao ^a^, Yi Chen ^a^, Zongping Zhu ^a^, Jiao Chen ^a^, Tianhui Gao ^a^,

Boonjai Limsila ^b^, Yenchit Techadamrongsin ^b^, Lei Wang ^c^, Jiali Yu ^a^, Chaomei Fu ^a,*^ , Rui Li ^a,*^

^a^ College of Pharmacy, State Key Laboratory of Characteristic Chinese Drug Resources in Southwest China, Chengdu University of Traditional Chinese Medicine, Chengdu, P.R. China.

^b^ Institute of Thai-Chinese Medicine Department of Thai Traditional and Alternative Medicines, Ministry of Public Health，Bangkok 11000, Thailand

^c^ Sichuan Provincial Orthopedic Hospital, 610041, China

*Corresponding authors:

Dr. Rui Li, E-mail: lirui@cdutcm.edu.cn

Prof. Chaomei Fu, E-mail: chaomeifu@126.com

^1^ These two authors contributed equally to this paper

**Chromatographic condition**

Waters 2695 HPLC system (Waters Corp., Milford, Massachusetts, USA) used in this experiment consists of an autosampler, quaternary pump, photo-diode array detector and a column temperature controller. System control and data analysis were processed with Waters Empower 2 software. The chromatographic separation was performed on a Agilent Zorbax SB-C18(150 mm×4.6 mm, 5μm) using 1% acetic acid aqueous solution (A, vol/vol), acetonitrile (B, vol/vol) and deionized water (C, vol/vol) as mobile phase at a flow rate of 1mL /min at 25 °C. The gradient program was set as **Supplementary Table 1.** The chromatogram was monitored at a wavelength of 214 nm and 420 nm throughout the experiment and the injection volume of each sample and standard solution was 5 μL. The HPLC mobile phase was prepared fresh daily, filtered through a 0.45μm membrane filter and then degassed before injected into the column.

**Supplementary Table 1.** Program of gradient elution

| *t*/min | mobile phase | | |
| --- | --- | --- | --- |
|  | A/% | B/% | C/% |
| 0~5 | 60~40 | 40~60 | 0 |
| 5~8 | 40~0 | 60~20 | 0~80 |
| 8~10 | 0 | 20~60 | 80~40 |
| 10~15 | 0 | 60~80 | 40~20 |
| 15~25 | 0 | 80~95 | 20~5 |
| 25~28 | 0 | 95~40 | 5~60 |

**Sample preparation**

According to the requirements of Chinese Pharmacopoeia 2015, 10 batches of vinegar-processed *C. phaeocaulis* powder were weighed (1.00 g) accurately, placed in a conical bottle, added 10 mL 80 % ethanol to the conical bottle and weighed it. These samples were placed overnight and processed by ultrasound (power 250 W, frequency 40 kHz) for 45 min. Then, these samples were taken out and cooled to room temperature, weighed it again and 80 % ethanol was used to make up for the loss. Each batch of sample needs to be filtered through a 0.22 μm membrane filter prior to injection.

**Standard compound**

Reference standards of bisdemethoxycurcumin, demethoxycurcumin, curcumin, curdione, curcumol, germacrone, and β-elemene were purchased from Sichuan Weikeqi Biological Technology Co., Ltd. (purity > 98%; Sichuan, China). HPLC of mixed reference substance is shown in **Supplementary Figure 1.**

**
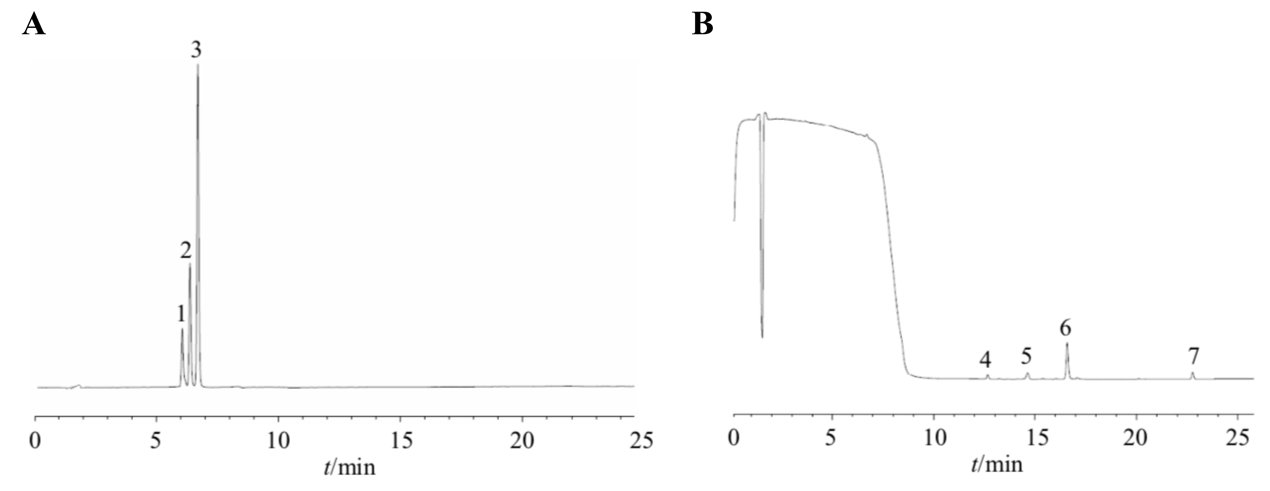
**

**Supplementary Figure 1.** HPLC of mixed reference substance (1: bisdemethoxycurcumin, 2: demethoxycurcumin, 3: curcumin, 4: curdione, 5: curcumol, 6: germacrone, 7: β-elemene)

The contents of bisdemethoxycurcumin, demethoxycurcumin, curcumin, curdione, curcumol, germacrone, and β-elemene were 0.00232, 0.05966, 0.2724, 0.3869, 1.2613, 0.7293, and 1.4212 mg/g, respectively. All the index compounds in vinegar-processed *C. phaeocaulis* samples in this study met the demand of the Pharmacopoeia of the People’s Republic of China, 2015 Edition.
